# [Retracted] Protective effect of microRNA‑381 against inflammatory damage of endothelial cells during coronary heart disease by targeting CXCR4

**DOI:** 10.3892/mmr.2024.13284

**Published:** 2024-07-08

**Authors:** Yimin Li, Jin Huang, Hong Yan, Xiangyu Li, Chang Ding, Qian Wang, Zhiping Lu

Mol Med Rep 21: 1439–1448, 2020; DOI: 10.3892/mmr.2020.10957

Following the publication of this paper, it was drawn to the Editor's attention by a concerned reader that the certain of the cell proliferation assay data shown in Fig. 4C on p. 1444 were strikingly similar to data appearing in different form in another article written by different authors at different research institutes, which had already been submitted for publication [Shi N, Shan B, Song Y, Chu H and Qian L: Circular RNA circ-PRKCI functions as a competitive endogenous RNA to regulate AKT3 expression by sponging miR-3680-3p in esophageal squamous cell carcinoma. J Cell Biochem 120: 10021–10030, 2019].

Owing to the fact that the contentious data in the above article were already under consideration for publication prior to its submission to *Molecular Medicine Reports*, the Editor has decided that this paper should be retracted from the Journal. The authors were asked for an explanation to account for these concerns, but the Editorial Office did not receive a reply. The Editor apologizes to the readership for any inconvenience caused.

